# Neutral Sphingomyelinase-2 (NSM 2) Controls T Cell Metabolic Homeostasis and Reprogramming During Activation

**DOI:** 10.3389/fmolb.2020.00217

**Published:** 2020-09-04

**Authors:** Maria Nathalia De Lira, Sudha Janaki Raman, Almut Schulze, Sibylle Schneider-Schaulies, Elita Avota

**Affiliations:** ^1^Institute for Virology and Immunobiology, University of Würzburg, Würzburg, Germany; ^2^Theodor-Boveri-Institute, Biocenter, Würzburg, Germany; ^3^Division of Tumor Metabolism and Microenvironment, German Cancer Research Center, Heidelberg, Germany

**Keywords:** neutral sphingomyelinase-2, T cell receptor, Seahorse XF, oxidative phosphorylation, ATP-adenosine triphosphate, Mitochondria

## Abstract

Neutral sphingomyelinase-2 (NSM2) is a member of a superfamily of enzymes responsible for conversion of sphingomyelin into phosphocholine and ceramide at the cytosolic leaflet of the plasma membrane. Upon specific ablation of NSM2, T cells proved to be hyper-responsive to CD3/CD28 co-stimulation, indicating that the enzyme acts to dampen early overshooting activation of these cells. It remained unclear whether hyper-reactivity of NSM2-deficient T cells is supported by a deregulated metabolic activity in these cells. Here, we demonstrate that ablation of NSM2 activity affects metabolism of the quiescent CD4^+^ T cells which accumulate ATP in mitochondria and increase basal glycolytic activity. This supports enhanced production of total ATP and metabolic switch early after TCR/CD28 stimulation. Most interestingly, increased metabolic activity in resting NSM2-deficient T cells does not support sustained response upon stimulation. While elevated under steady-state conditions in NSM2-deficient CD4^+^ T cells, the mTORC1 pathway regulating mitochondria size, oxidative phosphorylation, and ATP production is impaired after 24 h of stimulation. Taken together, the absence of NSM2 promotes a hyperactive metabolic state in unstimulated CD4^+^ T cells yet fails to support sustained T cell responses upon antigenic stimulation.

## Introduction

Neutral sphingomyelinase-2 (NSM2) is a sphingomyelin phosphodiesterase encoded by *SMPD3* gene which generates ceramides at the neutral pH optimum. It was first isolated from rat brain as an enzyme predominantly bound to the membranes (Liu et al., 1998). NSM2 activity is important for bone development and mineralization (Aubin et al., 2005; Stoffel et al., 2005), takes part in cellular stress responses or cytokine-mediated inflammation (IL1-β, TNF-α, IFN-γ), and also occurs after engagement of TNFR1, CD95, CD40, and TCR (Tonnetti et al., 1999; Airola and Hannun, 2013; Mueller et al., 2014; Shamseddine et al., 2015). NSM2 is bound to the cytosolic plasma membrane leaflet via N-terminal hydrophobic segments and generates ceramides there (Hinkovska-Galcheva et al., 1998; Tani and Hannun, 2007). Local reduction of sphingomyelin by sphingomyelinase activity results in increase of ceramides and generation of cholesterol which is free from stable interaction with sphingomyelin, possibly modifying membrane microdomain properties and performance in signal initiation. We and others found that NSM2-deficient cells have decreased plasma membrane ceramide levels and deregulated cholesterol homeostasis resulting in increased intracellular and plasma membrane accumulation of cholesterol (Qin et al., 2012; Bortlein et al., 2019).

When compared to those measured in brain or liver, expression levels of NSM2 in T-cells are rather low (Hofmann et al., 2000). Nevertheless, NSM2 activity proved to have a substantial impact on T-cell cytoskeleton dynamics, morphological polarization, and migration toward chemotactic signals, and, most importantly, for the optimal performance of TCR signaling (Gassert et al., 2009; Collenburg et al., 2017; Börtlein et al., 2018). Our more recent studies identified the TCR/NSM2/PKCζ pathway as crucial for TCR signal amplification and sustainment especially at low doses of stimulation (Börtlein et al., 2018). At a cellular level, NSM2-driven ceramide production essentially regulated PKCζ - dependent microtubule-organizing center (MTOC) dynamics as required for recycling and sustained supply of TCR signaling components to the plasma membrane at the immune synapse. Most importantly, NSM2 activity was also required for posttranslational modifications of tubulin such as acetylation and detyrosination which regulate its stability and microtubule polymerization. While these studies clearly support the importance of NSM2 in stimulated T cell response, they did not address a potential impact of the enzyme on sphingolipid homeostasis in T cells and, subsequently, on T cell metabolism.

T-cells undergo adaptive metabolic changes upon exit from quiescence, activation, and differentiation. Metabolic adaptation is decisive for the functional outcome of immune responses (Jung et al., 2019). In naïve T-cells, lymphatic S1P promotes mitochondria function and oxidative phosphorylation OXPHOS is the main source for ATP production while glycolytic activity is marginal (Pearce et al., 2013; Mendoza et al., 2017). Upon T-cell activation glucose, amino acid metabolism and OXPHOS are upregulated as is glycolysis which is referred to as glycolytic switch (Geltink et al., 2018). Along with boosting glycolysis, activated T cells actively restrain the oxidation of amino acids and lipids to produce ATP, while these substrates then rather serve as building blocks to support proliferation and cellular growth (Bauer et al., 2004). Signaling of the mechanistic target of rapamycin complex-1 (mTORC1) is essential for naive T-cell exit from quiescence, mitochondrial biogenesis, and activation of one-carbon metabolism (Yang et al., 2013; Ron-Harel et al., 2016). Maintenance of mitochondria membrane integrity and function of electron transport chain (ETC) during activation is crucial for T-cell effector function, and this depends on both proteins and lipids (Schenkel and Bakovic, 2014; Tarasenko et al., 2017), for example, mitochondria membrane protein voltage-dependent anion-selective channel 1 (VDAC1) functions in the metabolic cross-talk between mitochondria and cellular energy production (Shoshan-Barmatz et al., 2017). The exclusively mitochondrial membrane phospholipid cardiolipin CL is an essential component of mitochondria membrane and regulates mitochondria membrane potential and structural architecture. Deregulation of CL and cholesterol levels in mitochondria have been implicated in several human diseases, such as Barth syndrome and Niemann–Pick C1 disease (Barth et al., 1983; Porter et al., 2010).

The importance of sphingolipid metabolism in sustaining mitochondria functionality has been documented for cells of non-hematopoietic origin. Mitochondrial neutral sphingomyelinase activity and ceramides contribute to the damage of mitochondrial integrity and impaired OXPHOS in the heart reperfusion damage model (Ramirez-Camacho et al., 2016). Neutral sphingomyelinase in skeletal muscle myotubes inhibits ATP production and mitochondrial gene expression and enhances fatty acid-induced lipotoxicity (Verma et al., 2014). Similarly, in astrocytes ceramides regulate mitochondrial ATP release (Kong et al., 2018). Recent publication suggests that acid sphingomyelinase (ASM) regulates mitochondrial biogenesis and energy metabolism in ASM-deficient Niemann–Pick A/B patient fibroblasts (Yambire et al., 2019).

Little is known about the role of sphingomyelinases in the regulation of T-cell metabolic activity. Pharmacological inhibition of ASM was associated with impaired Akt/mTOR pathway activation in α-CD3/α-CD28-stimulated human naive, memory, and Th17-differentiated CD4^+^ T-cells (Bai et al., 2015). This study did, however, not address the role of sphingomyelinases or the impact of ceramides on metabolic activity in these cells.

Here we show the accumulation of intracellular mitochondrial ATP in peripheral blood human primary CD4^+^ T-cells in a NSM2- and tubulin dynamics-dependent manner. Quiescent NSM2-deficient T-cells revealed heightened basal metabolic activity as manifested by increased total cellular ATP levels and upregulated glucose uptake and glycolysis already prior to stimulation. This hyperactive metabolic state of unstimulated CD4^+^ T-cells supported robust early TCR-mediated mTOR/S6 signaling and metabolic switch immediately after α-CD3/α-CD28 stimulation. Accordingly, glycolysis and to a lesser extent OXPHOS were enhanced within the first 2 h after TCR/CD28 stimulation. However, 24 h poststimulation CD4^+^ T-cells lacking NSM2 activity became exhausted as measured by decreased mTOR/S6 signaling and OXPHOS and ATP production, resulting in impaired proliferation. In summary, we show that NSM2 activity supports sustained mitochondria function and energy production in stimulated CD4^+^ T-cells. Data presented here show a functional link between NSM2 and T-cell energy metabolism and contributes to the general understanding of molecular mechanisms involved in sphingomyelinase-dependent regulation of T-cell functions.

## Materials and Methods

### Ethics Statement

Primary human cells were obtained from the Department of Transfusion Medicine, University of Wuerzburg, and analyzed anonymously. All experiments involving human material were conducted according to the principles expressed in the Declaration of Helsinki and ethically approved by the Ethical Committee of the Medical Faculty of the University of Wuerzburg.

### Cell Culture

Primary human peripheral blood mononuclear cells (PBMCs) were isolated from healthy donors by Histopaque-1077 gradient centrifugation. CD4^+^ T cells (purity > 95%) were enriched from the peripheral blood lymphocytes (PBLs) using MagniSort^TM^ Human CD4 T cell Enrichment Kit (Invitrogen by Thermo Fisher Scientific) and maintained in RPMI 1640/10% FCS. CD4^+^ T cells were isolated from at least 50 different healthy donors to perform the experiments. CRISPR/Cas9-edited Jurkat cells deficient for NSM2 (ΔNSM) (Börtlein et al., 2018) cells were cultured in RPMI/10%FBS.

### T-Cell Nucleofection and NSM Inhibition

Neutral sphingomyelinase (NSM) pharmacological inhibition was achieved by incubating primary CD4^+^ T-cells with 1 μM GW4869 (SMPD2- and SMPD3-specific inhibitor, kindly provided by Cristoph Arenz) or with 1.5 μM ES048 (Collenburg et al., 2017) 2 h before or 2 h after α-CD3/CD28 stimulation. Nucleofection of human T cells was performed according to the manufacturer’s protocol (Lonza). For the silencing of NSM2, human T cells were nucleofected twice with a 2-day interval with 400 pmol siRNA targeting human *SMPD3* (NSM2) (5′-UGCUACUGGCUGGUGGACC-3’, 5′-GG CUCCACCAGCCAGUAGCA-3′) or, for control, a non-targeting siRNA (Sigma-Aldrich). Cells were harvested at day 5 and analyzed for viability and NSM activity. Cell viability was assessed by exclusion of propidium iodide (PI)-positive cells by flow cytometry according to the protocol of the manufacturer (BioLegend).

Knockdown efficiencies were analyzed by RT-PCR as previously described (Börtlein et al., 2018). Total RNA from 2 × 10^6^ NSM2 and control siRNA-nucleofected T cells was isolated 5 days post-transfection using TRIzol Reagent (Life Technologies) after the manufacturer’s protocol. cDNA was synthesized using First-Strand cDNA Synthesis Kit (Thermo Fisher Scientific) and used for PCR performed with Phusion Polymerase (Thermo Fisher Scientific) and NSM2 cDNA specific PCR primer: forward 5′ GCAGCTTCAAGTGTCTCAACAG 3′, reverse 5′ GTAGTGGGTGAACAGGGAGTGT 3′.

On average, knockdown efficiencies were higher than 50% at the enzyme activity level. NSM activity was determined as previously described (Tonnetti et al., 1999; Mueller et al., 2014) with modifications. 1–2 × 10^6^ T cells were disrupted by freeze/thawing (−80°C) in NSM lysis buffer without detergents (20 mM Tris pH 7.4, 10 mM β-glycerophosphate, 5 mM DTT, protease inhibitors). Nuclei were removed by centrifugation for 5 min at 1600 rpm. Post-nuclear homogenates were used directly for analysis of NSM silencing efficiency in unstimulated cells or aCD3/CD28 stimulated for different time points. Cell extracts were incubated with 1.35 mM HMU-PC (6-hexadecanoylamino-4-methylumbelliferyl-phosphorylcholine, manufactured by Biosynth Carbosynth) in NSM lysis buffer at 37°C for 17 h (final volume 30 μl). Fluorescence reading was performed using excitation at 404 nm and emission at 460 nm according to the manufacturer’s protocol. The assay determines the activity of all cellular neutral sphingomyelinases functional at neutral pH, which is optimal for enzymatic activity.

### T Cell Proliferation Assay

Triplets of 1 × 10^5^ T cells were stimulated with α-CD3- (clone UCHT-1) alone or together with a CD28-specific antibody (clone CD28.2) (1 μg/ml) (both: Becton-Dickinson Biosciences Pharmingen) on ice for 20 min, subsequently transferred to 96 well plates pre-coated with 25 μg/ml α-mouse IgG (Dianova) (1 h at 37°C). T cells were stimulated for 5 days including a final 24 h labeling period ([^3^H]-thymidine (Amersham)), and proliferation was analyzed using a microplate scintillation counter. Alternatively, T CD4^+^ T cells were labeled with CFSE (Affymetrix/eBioscience; 5 μM, 10 min) and proliferation was analyzed by flow cytometry.

### Generation of Mitochondria-Targeted GFP Cell Lines

CTRL and ΔNSM Jurkat cells stably expressing mitochondria-targeted GFP were created by using a lentivirus vector expressing a pre-sequence of the CoxV GFP (kindly provided by V. Kozjak-Pavlovic) for the Jurkat cell transduction.

### Incubation of Cells With Exogenous C16 Ceramide

Jurkat cell culture was supplemented with C16 ceramide as described previously (Collenburg et al., 2016; Börtlein et al., 2018). Shortly, a total of 5 × 10^7^ Jurkat-CTRL or Jurkat-ΔNSM cells were extensively washed and resuspended in RPMI/2% FBS containing 25 μM C16-ceramide (Avanti Polar Lipids), incubated overnight at 37°C, and washed three times with PBS before performing flow cytometry or mitochondria isolation. Cell viability was assessed by exclusion of propidium iodide (PI) and Annexin V-positive cells by flow cytometry according to the protocol of the manufacturer (BioLegend).

### Mitochondria Isolation

Human primary T-cells were left untreated or treated with 1.5 μM ES048 for 2 h followed by incubation in a medium containing DMSO solvent control or 10 μM nocodazole for 1 h. Subsequently, mitochondria were isolated as previously described (Chen et al., 2016) with slight modifications. Cells were harvested, pelleted, and resuspended in cold mitochondrial isolation media (MIM, 300 mM sucrose, 10 mM HEPES, 200 μM EDTA, and 1 mg/mL BSA, pH 7.4) at a ratio of 5 × 10^7^ per 500 μL of MIM. Cells were homogenized with a plastic homogenizer for 10 strokes. After homogenization, samples were centrifuged at 1600 RPM at 4°C for 7 min to separate the mitochondria from the remaining cellular material. The supernatant was collected and centrifuged at 13,000 RPM at 4°C for 10 min to obtain the mitochondrial pellet. The mitochondrial pellet was washed with cold-BSA free MIM followed by ATP measurement.

### ATP Measurement

Total cellular or mitochondria ATP levels were measured by an ATP determination kit (Molecular Probes). Human CD4^+^ T cells were pretreated or not with 1 μM oligomycin, 25 mM 2-DG, or both for 20 min or with Nocodazole 10 μM for 1 h. At the end of the incubation period, cells were collected and washed once with ice-cold PBS. Cells or isolated mitochondria were permeabilized with 1% Triton X-100 and the supernatant was kept on ice, or frozen in −80°C until the quantification of ATP. A concentration of 1 ng protein was used for the ATP assay. The luminometer readings were performed using the Centro XS3 LB960 microplate reader.

### Metabolic Profiling by Flow Cytometry

To determine the glucose uptake, human CD4^+^ T cells were incubated in PBS with 50 μM 6-deoxy-6-[(7-nitro-2,1,3-benzoxadiazol-4-yl)amino]-D-glucose (6-NBDG) for 60 min at 37°C. MitoTracker Green and MitoTracker Red (Invitrogen) were used to determine mitochondrial mass and membrane potential, respectively. Human CD4^+^ T cells were collected and incubated with 200 nM MitoTracker Green or Red for 30 min at 37°C. To determine the levels of the Glucose Transporter, Glut1 and mTOR activity cells were fixed with 4% paraformaldehyde at room temperature for 20 min, washed with FACS buffer (PBS/5 g BSA/2%NaN) and stained for phospho-S6 ribosomal protein (Ser235/236, Cell Signaling Technology) or anti-Glucose Transporter Glut1 antibody (ab15309) for 1 h at 4°C, antibodies were diluted in PBS with 0.3% saponin for intracellular staining, washed and incubated with secondary antibody Alexa488 for 30 min at 4°C (Invitrogen). Cells were washed with FACS buffer and analyzed using a BD FACSCalibur (BD Biosciences).

### Metabolic Flux Analyses

The metabolic flux analyses were performed using a Seahorse XF96. Extracellular acidification (ECAR) and oxygen consumption rates (OCR) were measured to assess the glycolytic activity and mitochondrial function of human CD4^+^ T-cells or Jurkat cells deficient or not for NSM2. Cells were seeded at a density of 1 × 10^6^ cells/well (8 wells per condition). To assess the metabolic response upon stimulation, we performed 4 basal measurements followed by injection with α-CD3/CD28 10 μg/ml and 10 postinjection measurements. The glycolytic stress test consisted of 3 distinct injections. The cells were first incubated in basic medium without glucose before receiving the first injection of 10 mM glucose to assess the basal glucose utilization; the second injection 1 μM oligomycin, a chemical responsible for blocking the mitochondrial ATP production, forcing the cells to utilize glycolysis; and the third injection 50 mM 2-DG, a glucose analog that blocks the glycolysis. The mitochondria stress test also consisted of 3 distinct injections. The cells were kept in full nutrient containing medium (250 mM glucose, 1 mM pyruvate, 2 mM L-Glutamine, pH 7.4) before receiving the first injection of 1 μM oligomycin, a chemical responsible for blocking the mitochondrial ATP production; the second injection 1.5 μM FCCP, a chemical that leads to the uncoupling of the mitochondrial membrane to assess the maximal cellular respiration; and the third injection a combination of 100 μM Rotenone and 1 μM Antimycin A, which block complexes I and III, respectively, in the electron transport chain.

### Western Blot Analysis

Human CD4^+^ T Cells were collected after the different time points of α-CD3/28 co-stimulation. Proteins were extracted from cultured cells, and all samples were normalized to 30 μg. Cellular proteins were separated by sodium dodecyl sulfate-polyacrylamide gel electrophoresis (SDS-PAGE). The following antibodies used in this study were purchased from Cell Signaling [Phospho-S6 ribosomal protein (Ser235/236) (pMTOR (Ser2448, D9C2), pAKT (Ser473, D9E), VDAC1/2 (cat. no. 4866), hexokinase II (clone C64G5)], or Santa Cruz Biotechnology [Aldolase A (C10), GAPDH (0411)]. The bands were visualized using SuperSignal West Pico PLUS detection reagent (Thermo Fisher Scientific), and the immunoblotting signals were quantified by densitometric scanning (ImageJ software 1.47v, National Institutes of Health, Bethesda, MD, United States).

### Immunostaining and Confocal Microscopy

To visualize the phosphorylation of mTOR, CD4^+^ T cells were incubated or not with ES048 for NSM inhibition and seeded in Lab-Tek^®^ II Chamber Slide System and stimulated with α-CD3/CD28-coated beads for different time points. Cells were washed once with PBS and fixed with 4% PBS for 30 min RT. After washing, cells were permeabilized using Triton 0.1% X-100 in PBS 5% BSA for 15 min. Cells were incubated with primary antibody Phospho-mTOR (Ser2448) (Cell Signaling) in PBS 5% BSA overnight at 4°C, washed 3 times, and cells were stained with α-rabbit Alexa488-conjugated secondary antibody (Invitrogen). To visualize the mitochondria, GFP cells were seeded in a Lab-Tek^®^ II Chamber Slide System and fixed with 4% PFA for 15 min and washed 3 times with PBS. After washing, the samples were mounted into slides. Samples were visualized by Confocal Laser Scanning Microscopy (CLSM) imaging performed using an LSM 510 Meta (Zeiss, Germany), equipped with an inverted Axiovert 200 microscope and a 40 × or 63 × EC Plan-Apo oil objective (numerical aperture 1.3 or 1.4, respectively) and laser lines 488. Image acquisition was performed with Zeiss LSM software 3.2 SP2.

### Image Analysis

Confocal images of cells were taken from different fields of view randomly selected across the entire coverslip area, and their mitochondrial morphology was analyzed using the semiautomated morphometric tool MiNA (Valente et al., 2017) within Fiji 50–100 cells were analyzed.

### Statistical Analysis

Data were analyzed with GraphPad Prism software (GraphPad San Diego, United States). Data shown were acquired in at least three independent experiments involving individual donors. For statistical analyses of data sets, unpaired Student’s *t*-test (^∗^*p* < 0.05, ns: non-significant) was used throughout the manuscript. Bars show standard deviations.

## Results

### NSM2 Regulates Metabolic Activity of Quiescent T-Cells

**FIGURE 1:**
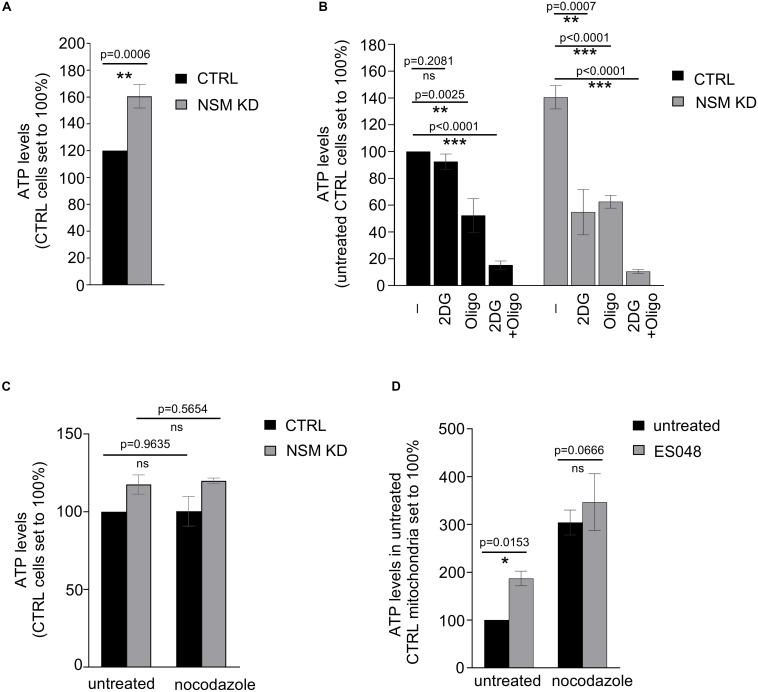
Glycolysis-dependent ATP production is upregulated in NSM2-deficient resting peripheral blood T-cells. **(A–C)** ATP was measured in the total lysates of CTRL and NSM2 siRNA transfected primary human CD4^+^ T-cells which were left untreated **(A)**, treated with 25 mM 2DG, 1 μM oligomycin or both for 20 min **(B)** or incubated with 10 μM nocodazole for 1 h. **(D)** PBLs were left untreated or were treated with 1.5 μM ES048 for 2 h followed by incubation with cell medium containing 10 μM nocodazole or not for 1 h. Mitochondria were isolated and ATP levels were analyzed. Data and standard deviations are shown from three independent donors normalized against untreated or CTRL siRNA-transfected cells (*n* = 3). *p*-values are shown on the top of significant (marked with asterisks: *p < 0.05, **p < 0.005, ***p < 0.0005) or not significant differences (ns).

Our previous study supported the importance of the NSM2 in human primary T cells in elevating threshold levels of T cell activation after TCR/CD28 co-stimulation: enhanced actin dynamics, Ca^2+^ mobilization, and general increase in tyrosine phosphorylation (Mueller et al., 2014). T cells deficient for NSM2 activity did also reveal enhanced basal activity of the protein kinase network prior to stimulation. Because signal transduction and protein kinase activation are ATP-consuming, energy-demanding processes, we reasoned that enhanced basal signaling in NSM2-deficient T cells might indicate deregulated energy metabolism in NSM2-deficient T-cells. To analyze whether NSM2 regulates ATP production in T cells, ATP concentrations were determined in whole-cell lysates of unstimulated human primary CD4^+^ T cells transfected with NSM2-specific or control siRNAs (further on named NSM KD or CTRL accordingly). NSM2-specific mRNA expression was nearly completely abolished whereas total activity of neutral sphingomyelinases was reduced by about 50% in NSM KD cells (Supplementary Figures S1A,B). Along with loss of NSM2 activity, ATP levels were significantly increased by about 40% as compared to CTRL cells (Figure 1A).

Next, we wanted to identify which metabolic pathway accounts for enhanced ATP levels in unstimulated NSM KD primary human T cells. ATP generated by oxidative phosphorylation (OXPHOS) in mitochondria is the main ATP source in quiescent T-cells (van der Windt and Pearce, 2012; Buck et al., 2015). This also applied to CTRL cells in our system in which inhibition of mitochondrial ATP production by oligomycin reduced ATP levels by about 50% while that of glycolysis by 2-deoxyglucose (2DG) did not significantly affect ATP levels (Figure 1B, left bars). Surprisingly, treatment of both oligomycin and 2DG reduced ATP levels to a comparable extent in NSM KD cells (Figure 1B, right bars), indicating that resting NSM2-deficient T cells are exploiting glucose as an ATP source additional to OXPHOS.

We and others demonstrated that NSM2 regulates ceramide generation and tubulin dynamics, which are both important for activity of mitochondrial voltage-dependent anion channel 1 (VDAC1) and mitochondrial ATP release in astrocytes (Börtlein et al., 2018; Kong et al., 2018). Expression levels of VDAC1 did not detectably differ in NSM KD and CTRL T cells (Supplementary Figure S2). To analyze the impact of tubulin assembly on ATP release in T cells, we elevated cytosolic tubulin by nocodazole in CTRL and NSM KD cells. Thereby, contact formation of tubulin with mitochondrial membranes is enforced, which was shown to block VDAC1-mediated ADP/ATP transport between mitochondria and cytosol (Kong et al., 2018). The nocodazole treatment did not affect total ATP levels in CTRL and NSM KD cells (Figure 1C). Next, we examined mitochondrial ATP transport channel functionality by measuring ATP levels in mitochondria isolated from primary T cells pretreated with a NSM2-specific inhibitor ES048 (Collenburg et al., 2017) which reduced NSM2 activity about 40% (Supplementary Figure S1B) or nocodazole. Inhibition of NSM2 as well as cytosolic tubulin elevation significantly enhanced mitochondria ATP content, indicating that both NSM2 and tubulin regulate ATP accumulation in T-cell mitochondria (Figure 1D).

**FIGURE 2:**
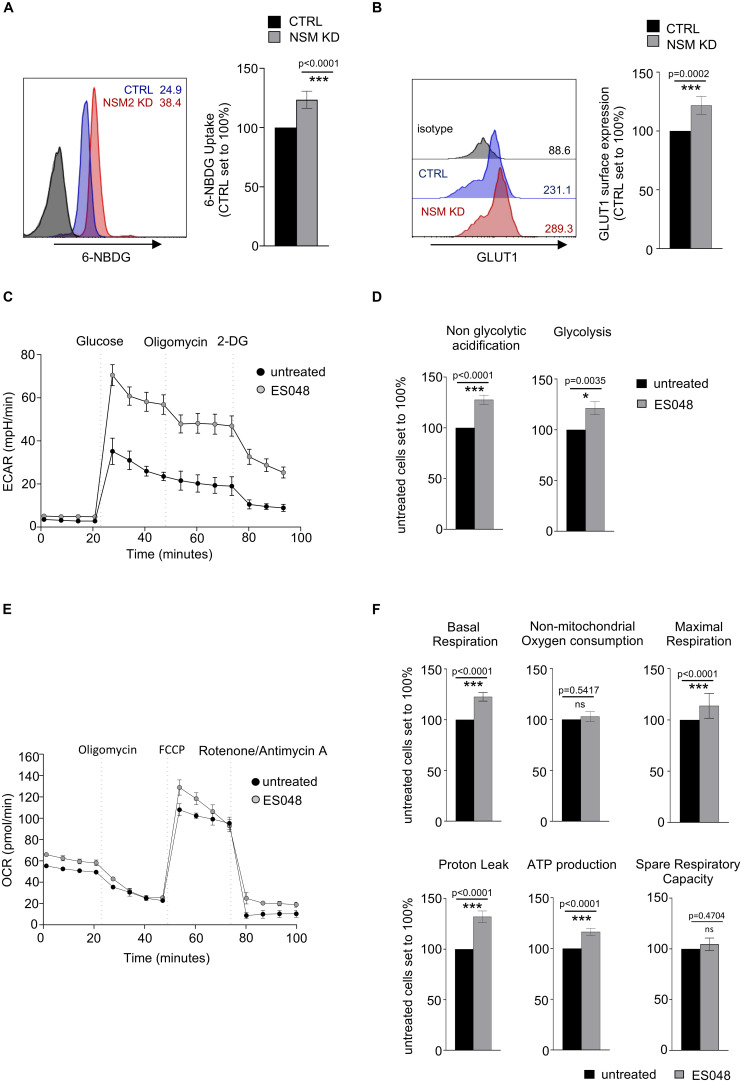
NSM2 deficiency promotes higher glycolytic and mitochondrial activity of resting T cells. **(A,B)** 6 NBDG uptake **(A)** and GLUT1 surface expression levels **(B)** were analyzed in CTRL and NSM2 siRNA-transfected primary human CD4^+^ T-cells by flow cytometry. One representative graph of each 6-NBDG uptake or Glut1 expression is shown (**A,B**; left graphs). Data obtained from three independent donors are summarized (**A,B**; right graphs). **(C–F)** CD4^+^ T cells were left untreated or pretreated with ES048 for 2 h and glycolytic **(C,D)** or mitochondrial stress tests **(E,F)** were performed and extracellular acidification rates (ECAR) and oxygen consumption rates (OCR) were detected using XF96 Seahorse analyzer. One representative graph for each ECAR **(C)** or OCR **(E)** measurement is shown. Levels of non-glycolytic acidification and glycolysis are shown in **(D)**. Basal respiration, non-mitochondrial oxygen consumption, maximal respiration, proton leak, ATP production, and spare respiratory capacity are shown in **(F)**. Data and standard deviations are shown from three independent experiments (*n* = 3). Measurements were normalized against untreated or CTRL siRNA-transfected cells which were set to 100%. *p*-values are shown on the top of significant (marked with asterisks) or not significant differences (ns).

To investigate whether enhanced glucose uptake supports generation of more ATP in resting NSM2-deficient cells, uptake of fluorescent non-hydrolyzable glucose analog 6-NBDG into these was monitored by flow cytometry. Both genetic (Figure 2A) and pharmacological (Supplementary Figure S3A). NSM2 inhibition significantly enhanced glucose uptake in primary CD4^+^ T cells. In agreement with Glut1 being a key receptor for glucose uptake in T cells (Macintyre et al., 2014), Glut1 expression levels on NSM2-deficient cells significantly exceeded those measured on control cells (Figure 2B and Supplementary Figure S3B). Interestingly, total Glut1 expression levels were elevated only upon genetic, but not upon pharmacologic, NSM2 inhibition (Supplementary Figure S3C). Glucose is a regulator of gene transcription and particularly regulates expression of glycolytic genes (Meugnier et al., 2007). We wanted to investigate if enhanced glucose uptake in NSM2-deficient unstimulated T cells affects expression of glycolytic proteins. Therefore, we analyzed expression of hexokinase II, aldolase A, and glyceraldehyde 3-phosphate dehydrogenase (GAPDH) as examples for glycolytic enzymes. In contrast to the enhanced uptake of glucose analog 6-NBDG, we did not observe any changes in the expression of glycolytic pathway proteins tested in our study (Supplementary Figure S3D).

Hypothesizing that enhanced glucose uptake might translate into enhanced glycolytic activity in resting T cells after pharmacologic NSM2 inhibition, we measured extracellular acidification rate (ECAR) of CD4^+^ T cell cultures exposed to ES048 or solvent, respectively, upon glycolytic stress conditions. For this and all subsequent experiments involving the Seahorse flux analyzer, we had to rely on pharmacologic inhibition of NSM by ES048 rather than siRNA ablation after which substantial cell death due to nucleofection of both specific and unspecific siRNA rendered analyses not satisfactory. In solvent-treated cells, glucose injection substantially raised ECAR, which was slightly sensitive to inhibition of mitochondrial ATP synthase by oligomycin treatment and highly sensitive to inhibition of glucose hexokinase by 2-DG (Figure 2C), confirming that ECAR measured in this assay reflects glycolysis. Observed T cell glycolytic response to saturating concentrations of glucose was in agreement with the published observations that general glycolytic performance of naive and unstimulated T cells is low and does not improve after oligomycin treatment (Pearce et al., 2013; Buck et al., 2015). ES048 treatment significantly enhanced ECAR in primary CD4^+^ T cells (Figures 2C,D), indicating that enhanced glucose uptake fuels enhanced the glycolytic rate, which serves as additional ATP source in NSM2-deficient T cells.

Interestingly, non-glycolytic acidification, likely to be caused by respiratory CO_2_ generated by the tricarboxylic acid (TCA) cycle and converted to HCO_3_^–^ and H^+^, was also found to be enhanced in ES048-treated T-cells (Figure 2D), indicating that the TCA cycle feeding into mitochondrial ATP production by OXPHOS could be regulated by NSM2 activity. We therefore determined oxygen consumption rates (OCR) in solvent and ES048-treated CD4^+^ T cells employing a mitochondrial stress test. Basal respiration values measured in ES048-treated cells slightly exceeded those in solvent controls, indicating pronounced energetic demand in NSM2-deficient T cells (Figures 2E,F). More than 50% of basal respiration was linked to ATP production, and this was even more pronounced in ES048-treated cells (Figure 2F). Parameters of mitochondria functionality, maximal respiration and proton leak, were significantly increased after pharmacological inhibition of NSM2.

Altogether, data obtained in resting T cells suggest that NSM2 deficiency rather promotes mitochondrial function, while both NSM2 and depolymerized tubulin interfere with the intracellular distribution of mitochondria-generated ATP. Because ATP is trapped in mitochondria of NSM2-deficient T-cells, it may not fully satisfy cellular energy demands, which can be complemented by these cells upon activation of glycolysis, providing an additional ATP source.

### NSM2 Dampens Early Metabolic Responses to Antigenic Stimulation of T-Cells

**FIGURE 3:**
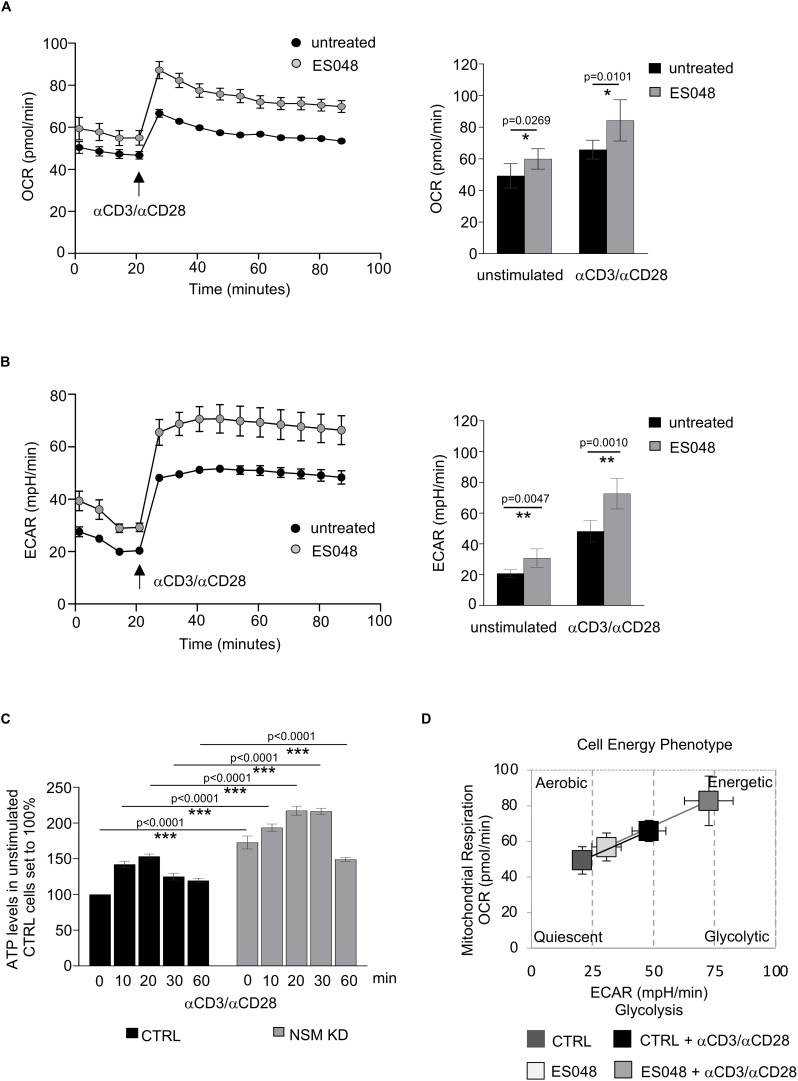
NSM2 suppresses overshooting early metabolic responses to TCR/CD28 stimulation. **(A,B)** CD4^+^ T cells were left untreated or treated with ES048 for 2 h followed by measurement of OCR **(A)** and ECAR **(B)** before and after CD3/CD28 injection. Maximal values for OCR or ECAR before and after α-CD3/CD28 injection are shown (**A,B**; right graphs). **(C)** ATP levels were quantified after α-CD3/CD28 co-stimulation of CD4^+^ T cells transfected with CTRL or NSM2-specific RNAs. Mean values of three independent experiments with standard deviations are shown. **(D)** Analysis of the cell energy phenotype in ES048-pretreated CD4^+^ T cells. Measurements were normalized against untreated cells or CTRL siRNA-transfected cells which were set to 100%. *p*-values are shown on the top of significant (marked with asterisks) or not significant differences (ns).

Previously, we showed that NSM2-deficient T-cells hyper-respond early after TCR/CD28 stimulation (Mueller et al., 2014). Assuming that this might rely on elevated metabolic activity of these cells, CD4^+^ T cells were exposed to the NSM inhibitor ES048 for 2 h in glucose and pyruvate containing medium, and OCR and ECAR were measured prior to and 1.5 h after co-stimulation. Both measured parameters were significantly increased in ES048-treated T cells before and after stimulatory antibody injection (Figures 3A,B). To further support this hypothesis, we co-stimulated NSM KD and CTRL cells with α-CD3/α-CD28 antibodies and measured ATP production over 1 h. ATP production was maximal after 20 min of stimulation in both cell cultures, with NSM KD cells producing significantly higher ATP levels at all-time points measured (Figure 3C). Moreover, pharmacologic NSM ablation promoted a higher energetic state in both quiescent and freshly stimulated T-cells (Figure 3D). Together, these data indicated that NSM2 indeed regulates threshold levels of T-cell metabolic activation.

### NSM2-Deficient T-Cells Fail to Sustain Metabolic Response to TCR Stimulation

**FIGURE 4:**
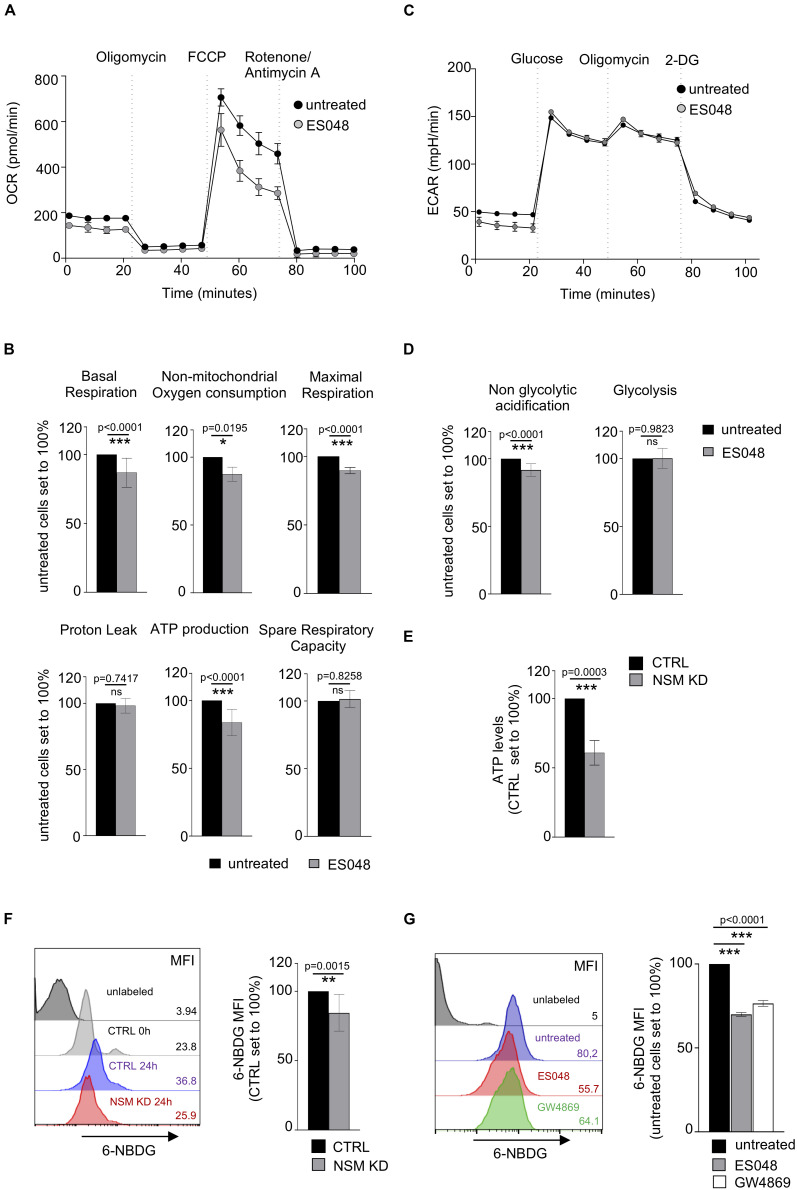
NSM2 activity fuels sustained mitochondria respiration, ATP production, and glucose uptake in TCR-stimulated T cells. CD4^+^ T cells were left untreated or treated with ES048 or transfected with NSM2-specific siRNA (NSM KD) and co-stimulated with α-CD3/CD28 for 24 h before experiments. Oxygen consumption rate (OCR) **(A)** and extracellular acidification rate (ECAR) **(C)** were measured in ES048-pretreated T-cells using an XF96 Seahorse metabolic flux analyzer. **(A)** Representative graph of the mitochondria stress test. **(B)** Analysis of basal respiration, non-mitochondrial oxygen consumption, maximal respiration, proton leak, ATP production, and spare respiratory capacity from independent experiments. **(C)** Representative graph of the glycolytic stress test. **(D)** Analysis of non-glycolytic acidification and glycolysis from independent experiments. **(E)** Cellular ATP levels were measured in lysates of α-CD3/CD28-stimulated CTRL and NSM KD cells. **(F,G)** Glucose analog 6-NBDG uptake was measured in CTRL and NSM KD cells **(F)** or in untreated or ES048- or GW4869-pretreated cells **(G)**. Mean values with standard deviations are shown. Measurements from three independent experiments were normalized against co-stimulated untreated or CTRL cells which are set to 100%. *p*-values are shown on the top of significant (marked with asterisks) or not significant differences (ns).

The mitochondrial metabolism is important for T-cell effector functions and proliferation where the TCA cycle contributes to the functionality of electron transport chain (ETC) in mitochondria upon activation (Bailis et al., 2019). To comparatively analyze the role of NSM2 in mitochondrial activity late after T cell activation, stress tests were performed in solvent or ES048-treated CD4^+^ T-cells 24 h after co-stimulation. Surprisingly, ES048 pre-exposure affected OCR profiles as seen by reduced maximal respiration after oligomycin and carbonyl cyanide 4-(trifluoromethoxy)phenylhydrazone (FCCP) injections (Figure 4A). Mitochondrial function was significantly impaired as measured by decrease in basal and maximal respiration as well ATP production (Figure 4B).

It has been shown that upon immune stimulation and activation, T-cells undergo metabolic switch by upregulating glycolysis with glucose metabolism being increased with onset of proliferation and sustained for the entire activation period in human T-cells (Renner et al., 2015). We analyzed expression levels of glycolytic enzymes: hexokinase II, aldolase a, and GAPDH, in CTRL and NSM KD cells after 24 and 48 h of α-CD3/CD28 stimulation. Both CTRL and NSM KD T cells upregulated the expression of those proteins upon stimulation (Supplementary Figure S3D) with NSM KD cells showing even enhanced protein levels after stimulation. To study whether elevated metabolic activity upon NSM inactivation extends beyond the early activation phase, we determined ECAR values in ES048-treated CD4^+^ T-cells 24 h after TCR/CD28 co-stimulation when T-cell blasts developed, and proliferation was initiated. In contrast to what has been seen prior to and early after co-stimulation, ECAR measurement profiles were comparable in NSM-deficient and -sufficient cells after 24 h of co-stimulation (Figure 4C), and analysis of glycolysis and non-glycolytic acidification levels revealed no significant differences (Figure 4D). Finally, total cellular ATP levels were decreased by about 40% in NSM2 siRNA-transfected KD cells (Figure 4E).

Seahorse technology was not applicable for T-cells nucleofected with NSM2 siRNA as the cell culture has enhanced amounts of dead cells after transfection. In addition to pharmacologic ablation of NSM in primary T-cells, Jurkat cells were available to us, in which NSM2 was genetically deleted by CrispR/Cas9. The resulting ΔNSM2 Jurkat cells revealed a 60% reduction of NSM activity (Supplementary Figure S1B). Similar as for ES048-treated T-cells, ΔNSM2 Jurkat cells which are permanently activated (independently of stimulation) showed strongly impaired mitochondria stress response whereas ECAR profiles did not show any significant differences (Supplementary Figures S4A,B).

Because glucose does provide not only energy through glycolysis but also building blocks for cellular maintenance and proliferation in activated T-cells, we next analyzed 6-NBDG glucose uptake in NSM-deficient and -sufficient T cells (van der Windt and Pearce, 2012). Surprisingly, genetic depletion of NSM2 by siRNA transfection or treatments with NSM inhibitors significantly reduced the uptake of 6-NBDG in CD4^+^ T-cells after 24 hours of TCR/CD28 co-stimulation (Figures 4F,G). The commercially available NSM inhibitor GW4869 has a similar inhibitory effect on NSM activity in stimulated T cells as compared to the ESO48 inhibitor used for metabolic flux analysis in this study (Supplementary Figure S1C). In line with glucose uptake being impaired, total Glut1 expression levels were also found reduced by 40–60% in NSM2-deficient cells (Supplementary Figure S4C).

Altogether, these data support a critical role of NSM2 in maintaining mitochondrial function late after co-stimulation in blast forming and proliferating T-cells.

### TCR-Activated mTOR Pathway Is Maintained by NSM2

**FIGURE 5:**
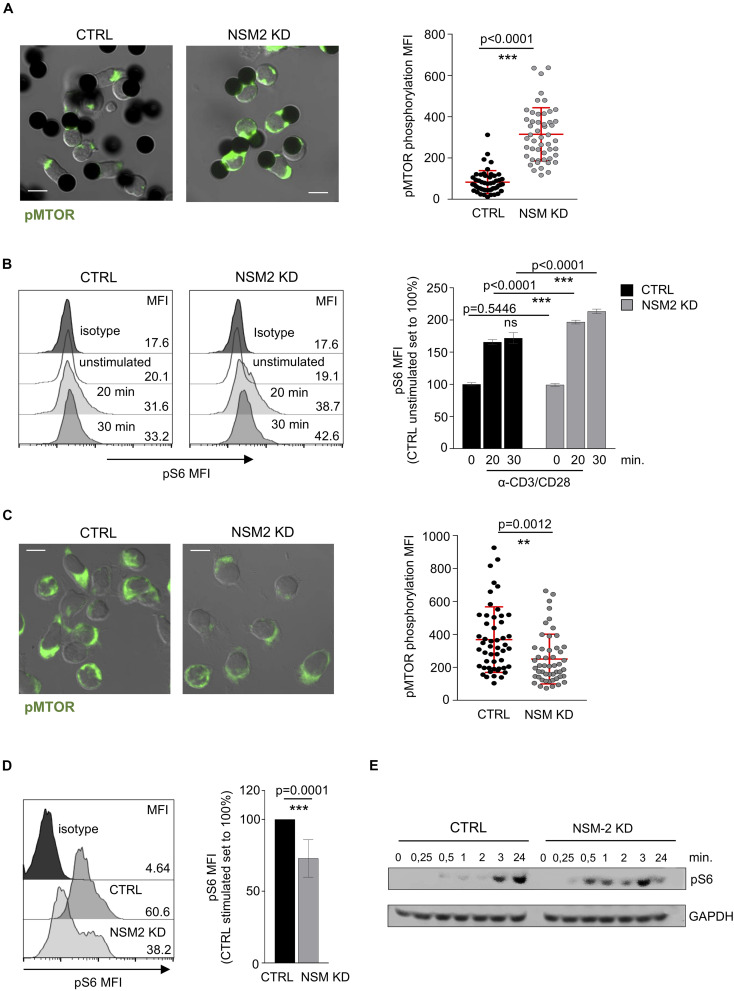
Sustained mTORC1 activity in activated T-cells is NSM2 dependent. **(A)** Representative fluorescence pictures (left panels) and quantification (right panel) of phosphorylated MTOR measured by confocal microscopy are shown for CTRL and NSM KD CD4^+^ T cells stimulated with α-CD3/CD28-coated beads for 20 min. **(B)** Mean fluorescence (MFI) of phosphorylated S6 was analyzed by flow cytometry in CTRL and NSM KD CD4^+^ T cells left untreated or co-stimulated with α-CD3/CD28 for 20 and 30 min. Representative flow cytometry graphs (left histograms) and quantification of pS6 (right graph) are shown. **(C)** Representative fluorescence pictures (left panels) and quantification (right panel) of phosphorylated MTOR measured by confocal microscopy are shown for CTRL and NSM KD CD4^+^ T cells co-stimulated with α-CD3/CD28 for 24 h. **(D)** Phosphorylation of S6 was analyzed by flow cytometry in CTRL and NSM KD CD4^+^ T cells left untreated or co-stimulated with α-CD3/CD28 for 24 h. Representative flow cytometry graph (left histograms) and quantification of pS6 (right graph) are shown. **(E)** Western blot analysis of pS6 in CTRL an NSM2 KD left untreated or co-stimulated with CD3/CD28 for different time points. Quantifications show mean values with standard deviations. Data were normalized against CTRL cells set to 100%. *p*-values are shown on the top of significant (marked with asterisks) or not significant differences (ns). Scale bar in fluorescence pictures: 10 μM.

As shown above, NSM2 appears to dampen mitochondrial activity in unstimulated or freshly co-stimulated cells, while it is obviously needed for sustainment of mitochondrial functions late after co-stimulation. The protein serine–threonine kinase mammalian/mechanistic target of rapamycin (mTOR) is a part of mTOR Complex 1 (mTORC1) which drives cellular proliferation and growth by upregulating the expression of metabolic enzymes and is a key regulator of mitochondrial oxidative metabolism (Lopez et al., 2019). We therefore analyzed the activation dynamics of mTOR in NSM2-deficient T-cells early and late after TCR/CD28 co-stimulation. Expression patterns of phosphorylated mTOR (pmTOR) were similar in CTRL and NSM KD CD4^+^ T-cells shortly after conjugation to α-CD3/α-CD28-coated beads with pmTOR localizing distally or in close proximity to the artificial synapses formed between cells and stimulatory beads. Interestingly, however, signal intensities of mTOR were significantly higher in NSM2-deficient cells (Figure 5A). As a readout of mTORC1 activity, we determined phosphorylation levels of its substrate ribosomal protein S6 kinase in response to co-stimulation over time by flow cytometry. In line with the high energetic state of NSM2-deficient cells early after activation, pS6 levels measured in NSM2 KD T-cells exceeded those in CRTL cells within 30 min of co-stimulation (Figure 5B). However, α-CD3/α-CD28-co-stimulated NSM KD T-cells had significantly lower pmTOR and pS6 levels later upon activation (Figures 5C,D). Similar, flow cytometry analysis of CD4^+^ T cells pretreated with NSM inhibitors ESO48 and GW4869 showed significant reduction of S6 phosphorylation levels after 24 h of stimulation (Supplementary Figure S5A). In agreement with the results obtained by flow cytometry, earlier rise, but not sustainment of pS6 levels in co-stimulated NSM2 KD as compared to CTRL T-cells, was also evident at the level of whole-cell lysates by Western blot analysis (Figure 5E).

The crucial role of NSM2 to support mTOR pathway and mitochondria functions in stimulated T cells seems to be in contrast with its dampening function in unstimulated or early-activated T cells. We compared NSM activity in untreated or NSM2 inhibitor-treated quiescent CD4^+^ T cells before and after 1 or 24 h of α-CD3/CD28 stimulation. Untreated control cells gradually increased NSM activity upon TCR/CD28 co-stimulation, reaching significantly higher levels 24 h poststimulation. As expected, ESO48- and GW4869-treated cells showed no significant increase in NSM activity upon stimulation (Supplementary Figure S1C). We wanted to find out how much impact NSM2 activity has on regulation of T cell metabolism at later stages of stimulation. To analyze that, we added NSM2 inhibitors ES048 and GW4869 to CD4^+^ T cells 2 h after α-CD3/CD28 stimulation and measured the uptake of glucose analog 6-NBDG and phosphorylation of S6 (Supplementary Figure S5B). The inhibition levels of 6-NBDG uptake and S6 phosphorylation were comparable to those measured in inhibitor pretreated cells (Figure 4G and Supplementary Figure S5A). The results indicate the crucial role of NSM2 in maintaining glucose uptake and mTOR pathway activity during the progression of T cell stimulation.

Metabolic switch requires activation of Akt kinase (Jones et al., 2019), which acts upstream of mTORC1 and promotes Glut1 expression and transport to the cell surface (Macintyre et al., 2014; Palmer et al., 2015). When compared in NSM KD or ES048-treated T-cells and their respective control cultures, levels of Akt phosphorylation (pAkt) were found similar in all cultures within the first hour of co-stimulation (Supplementary Figures S6A,B) correlating with the effective early metabolic switch shown above (Figure 3). Twenty four hours after stimulation, pAkt was barely detectable independently of NSM2 inactivation indicating that the PI3K/Akt pathway is available for mTOR pathway regulation only in the onset of T-cell activation. This indicated that differential activation of mTORC1 in NSM2-deficient T-cells is not mediated by Akt.

Altogether, analysis of TCR-dependent activation kinetics of the mTOR pathway indicated that NSM2 is important to sustain and keep this pathway active in proliferating cells.

### NSM2 Regulates Mitochondria Biogenesis and Membrane Potential in Jurkat Cells

**FIGURE 6:**
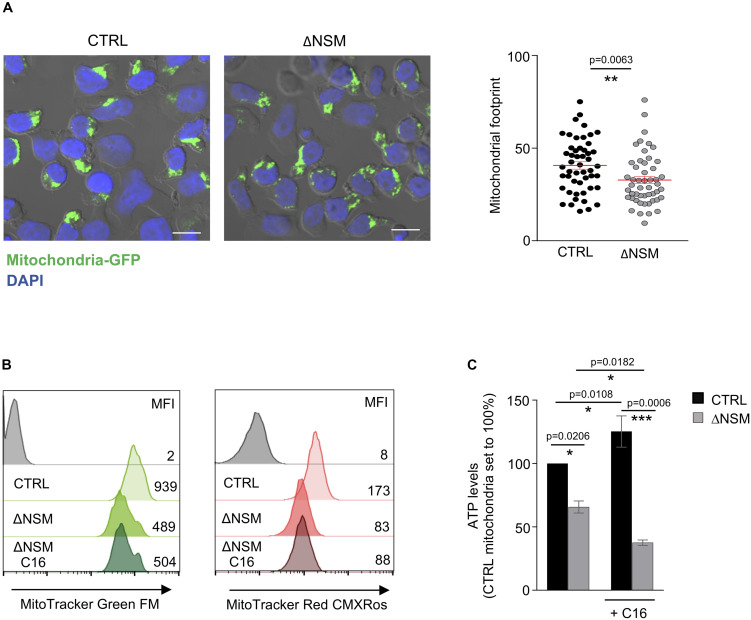
Upregulation of mitochondria mass and functionality is NSM2 but not ceramide dependent. **(A)** Representative mitochondria-GFP fluorescence pictures (upper panels) and quantification of the mitochondria footprints in individual cells (bottom graph) of stably transfected CTRL and ΔNSM Jurkat cell cultures are shown. Mitochondrial footprints (the areas or image volume consumed by mitochondria-GFP signal) (bottom panel) were calculated by morphometric ImageJ plug-in tool MiNA for analysis of confocal microscopy pictures. Mean values with standard deviations are shown. Scale bar 10 μM. **(B,C)** CTRL and ΔNSM Jurkat cells were cultured in medium supplemented or not with ceramide C16 overnight. Subsequently, mitochondria mass and membrane potential were analyzed by flow cytometry using MitoTracker Green and Red, respectively. Mean fluorescence (MFI) values are shown within the flow cytometry histograms. Alternatively, mitochondria were isolated and ATP levels were measured. *p*-value is shown on the top of significances (marked with asterisks).

As shown above, NSM2 was required to sustain respiration and ATP production of mitochondria in activated and proliferating T-cells. As suggested by previous studies (Buck et al., 2016), morphological changes of mitochondria are shaping T-cell metabolic reprogramming during TCR stimulation. We therefore stably expressed mitochondria-targeted GFP (Chowdhury et al., 2017) in CTRL and ΔNSM2 Jurkat cells to comparatively study mitochondria morphology. As revealed by confocal microscopy, mitochondrial footprints were significantly reduced in NSM2-deficient cells, which reflects a decreased size of these organelles (Figure 6A). A closer visual inspection of fluorescently tagged mitochondria did not show any obvious structural changes in ΔNSM2 Jurkat cells. Unfortunately, morphometric tool MiNA within Fiji (Valente et al., 2017) previously used for exact quantification of mitochondria structural details was not suitable to resolve the densely packed mitochondria in a quite narrow cytoplasmic space typical for T-cells.

To further investigate mitochondria size and membrane potential as parameters of their function, CTRL and ΔNSM2 Jurkat cells were labeled with MitoTrackers Green and Red, respectively. Recording membrane potential, MitoTracker Red fluorescence intensity was decreased in ΔNSM2 Jurkat cells, thereby indicating that NSM2 appears to be required for activities of respiratory complexes leading to effective ATP production (Figure 6B). In agreement with our observations made with regard to mitochondrial footprints (Figure 6A), mitochondrial size was also found to be reduced in NSM2-deficient cells by measurement of MitoTracker Green fluorescence intensity (Figure 6B). NSM2 activity is associated with ceramide generation at the plasma membrane of T cells. To investigate the impact of ceramides on mitochondria functionality, we incubated CTRL and ΔNSM2 Jurkat cells with exogenous C16 ceramide overnight and analyzed mitochondria size, membrane potential, and ATP levels. Our experimental setup of cell culturing with ceramide did not affect cell viability (Supplementary Figure S7), and previously we have shown that supplementing NSM2-deficient T cells with ceramides can rescue microtubule polarization which is impaired in ΔNSM2 Jurkat cells. Now we observed that ceramides alone did not improve mitochondria size, membrane potential, or ATP production (Figures 6B,C). Feeding ΔNSM2 cells with ceramides reduced mitochondria ATP levels even further down (Figure 6C), which possibly indicates improved ATP transport to the cytoplasm regulated by microtubules and rescued by ceramides. Results suggest that NSM2-dependent functionality of mitochondria in stimulated T cells is not solely dependent on ceramides. Moreover, the uptake and metabolic turnover of extracellularly added ceramides possibly do not exactly correspond to the localization, intracellular transport, or metabolism of NSM2-generated ceramides.

### NSM2 Activity Supports T Cell Expansion

**FIGURE 7:**
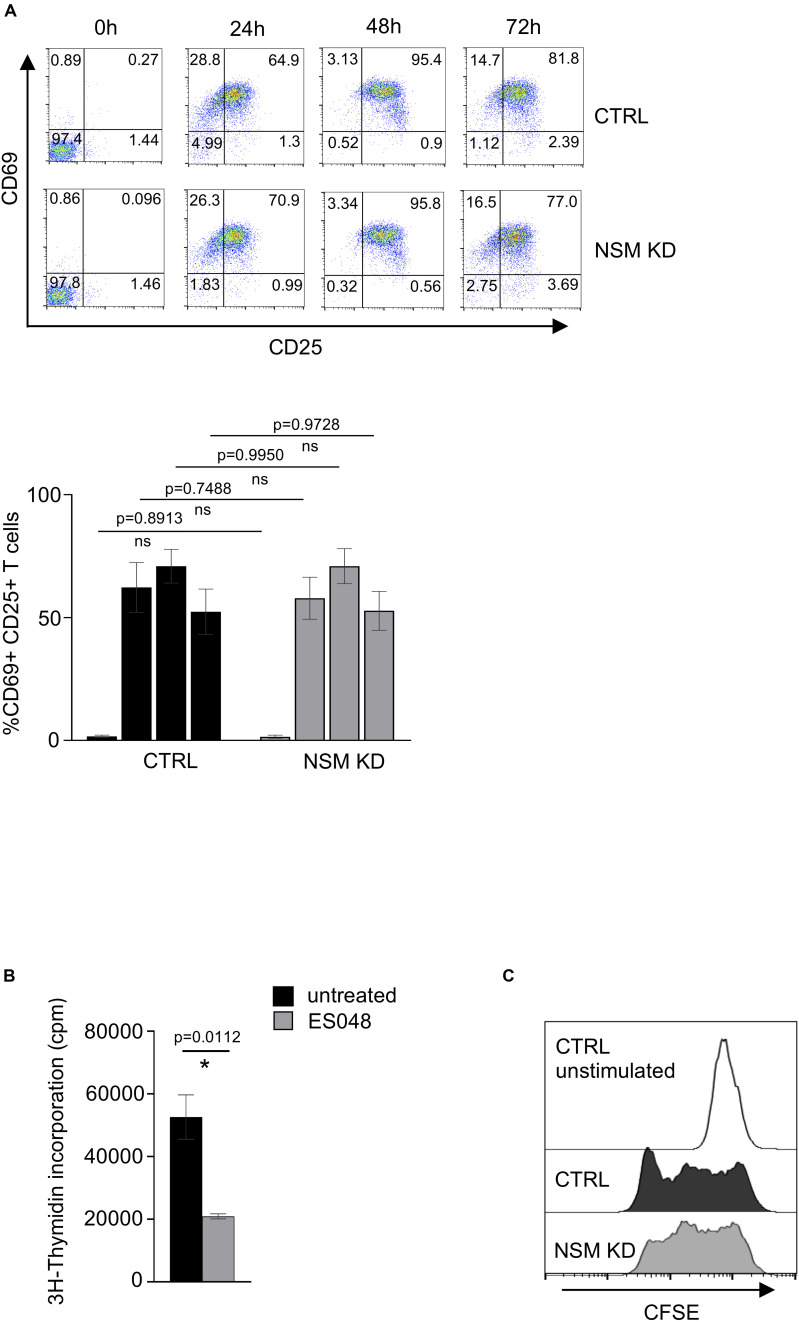
NSM2 activity supports T-cell expansion. **(A)** Expression of T-cell activation markers CD25 and CD69 were analyzed by flow cytometry in CD4^+^ CTRL and NSM KD T-cells cells after 24, 48, and 72 h of co-stimulation with α-CD3/CD28. Representative flow cytometry pictures (upper plots) and analysis of positive cells (bottom graph) are shown. **(B)** ^3^H-Thymidine incorporation in untreated and ES048-pretreated CD4^+^ T-cells after 5 days of α-CD3/CD28 co-stimulation. Mean values with standard deviations are shown. *p*-values are shown on the top of significant (marked with asterisk) or not significant differences (ns). **(C)** Proliferation of CFSE labeled CTRL and NSM KD CD4^+^ T-cells after 5 days of α-CD3/CD28 co-stimulation was analyzed by flow cytometry.

Enhanced mitochondria functionality upon T-cell activation is crucial for the increase of both OXPHOS and one-carbon metabolism, a serine-dependent pathway that regulates purine and pyrimidine biosynthesis necessary for cell proliferation (Tan et al., 2017). It was shown by several groups that the metabolites of the one-carbon pathway are upregulated in an mTOR-dependent manner and are important for T-cells to enter the S phase of the cell cycle but are not required for T-cell activation as they exit the quiescence. To study the impact of NSM2 depletion on cellular parameters of T cell activation, we analyzed upregulation of CD69 and CD25 marking early and late T-cell activation markers 24, 48, and 72 h after co-stimulation by flow cytometry. Maximal expression levels of both markers were detected on the surface of CTRL and NSM KD T-cells 48 h after co-stimulation with no NSM2 dependency being visible (Figure 7A). Inhibition of NSM2 activity, however, significantly impaired expansion of CD4^+^ T-cells 5 days after co-stimulation (Figure 7B). The proliferation profile of CFSE labeled and stimulated NSM KD or inhibitor-treated CD4^+^ T cells showed stalled CFSE dye dilution indicating that the speed of initial cell cycling is downregulated 5 days after a - CD3/CD28 stimulation (Figure 7C and Supplementary Figure S8A). In addition, the mTOR pathway can promote CD4^+^ T cell survival (Galgani et al., 2010). We tested the viability of neutral sphingomyelinase inhibitor treated or NSM KD CD4^+^ T cells after α-CD3/CD28 stimulation. The number of viable cells stayed similar for untreated and inhibitor-treated or NSM2 siRNA-transfected T cells independently of stimulation (Supplementary Figure S8B). Thus, NSM2 activity is required for CD4^+^ T cell expansion, but not for viability or upregulation of the measured activation markers.

## Discussion

Multiple studies support the role of NSM2 for a plethora of cellular processes including stress responses and cytokine-mediated inflammation in many cell types (Clarke et al., 2006; Airola and Hannun, 2013; Shamseddine et al., 2015), while its importance in T cell activation and metabolism is not well understood. We recently reported that phosphorylation levels of cellular proteins also including Src family protein tyrosine kinase Lck as well as membrane order are elevated in resting NSM2 knockdown human primary T-cells (Börtlein et al., 2018). Moreover, sphingomyelin and cholesterol contents in the plasma membrane of NSM2-deficient Jurkat cells were increased, suggesting that NSM2 activity might positively regulate membrane protein clustering and signaling (Bortlein et al., 2019). Here we show that resting human primary CD4^+^ NSM2 KD T-cells accumulate intracellular ATP which can be used to supply protein kinase signaling with metabolic energy (Figure 1A).

NSM2 deficiency increased ATP accumulation in T cell mitochondria (Figure 1D). This is in contrast to observations made in NSM2-deficient astrocytes where lack of intracellular tubulin-associated ceramides in mitochondria-associated membranes kept the voltage-dependent anion channel 1 (VDAC1) open thereby increasing mitochondria ATP release to the cytosol (Kong et al., 2018). This study established that ceramides are essential regulators of cytosolic tubulin association with VDAC1 regulating ATP transport. Recently, we have shown that at least in Jurkat cells ceramide levels decreased specifically in the plasma membrane, but not in organelle membranes upon NSM2 deficiency (Bortlein et al., 2019). We and others have demonstrated that NSM2-generated ceramides initiate PKCζ signaling at the plasma membrane and promote acetylation and polymerization of tubulin in several types of cells: stem cells, neuronal progenitors, and primary human T-cells (He et al., 2014; Börtlein et al., 2018). Based on these and our current results, we suggest that NSM2 activity supports tubulin polymerization, microtubule assembly, and disassociation from VDAC1 promoting ATP transport to support energy demands in cytoplasm. Here we propose that NSM2 deficiency in T-cells promotes depolymerization of tubulin, microtubule disassembly (Börtlein et al., 2018), and association with ceramides in mitochondria-associated membranes (generated NSM2 independently) thereby blocking VDAC1-mediated ADP/ATP transport between mitochondria and cytosol and accumulation of ATP in mitochondria.

We found that resting NSM KD CD4^+^ T-cells increase Glut1 expression, uptake of glucose analog 6-NBDG uptake and glycolytic activity probably to compensate for the lack of mitochondrial ATP in the cytoplasm (Figures 1D, 2A–D). This is in line with observations made earlier in myoblasts, where mito-energetic dysfunction triggered compensatory increase in glycolysis (Liemburg-Apers et al., 2015). The overall increase in ATP levels in resting NSM KD T-cells reflects increased activity of the glycolytic pathway (Figures 1B, 2C) and provides the basis for enhanced early activation. This was documented by rapid exit from quiescence upon TCR/CD28 co-stimulation: highly efficient and significantly enhanced upregulation of glycolysis, OXPHOS, and mTOR pathway (Figures 3, 5A,B).

In contrast, NSM2 downregulation did not affect glycolysis 24 h after T cell co-stimulation even though glucose uptake and Glut1 expression were significantly reduced (Figures 4C,F,G and Supplementary Figure 4C). Possibly, T cells compensate for lower glucose uptake in the absence of NSM2 activity in stimulated T-cells by metabolizing glycogen as shown for memory CD8^+^ T cells (Ma et al., 2018). Alternatively, uptake of the large neutral amino acids was shown to be important for metabolic switch in TCR-stimulated cells (Sinclair et al., 2013).

However, the higher initial mTOR and OXPHOS activation levels observed in NSM2-deficient cells were not contained at a later stage of TCR/CD28 engagement and did not support optimal proliferation (Figures 4A, 5, 7B). As established, OXPHOS is needed for continuous and sustained proliferation of activated T-cells for at least 2 days after stimulation (Chang et al., 2013). Though dispensable for activation, the one-carbon metabolism connected to mitochondria functionality is important for T-cell proliferation (Ron-Harel et al., 2016; Ma et al., 2017; Tan et al., 2017). In line with these findings, mTOR pathway activation, mitochondria size, respiration, and ATP production were significantly decreased in NSM2-deficient cells 24 h after TCR/CD28 engagement resulting in impaired T-cell proliferation without affecting expression levels of activation markers CD25 and CD69 (Figures 4–7). Reduced cellular ATP levels late after activation in stimulated NSM KD T-cells correlated with the reduced mitochondria functionality (Figure 4). Finally, NSM2-deficient T-cells have less intracellular ATP available for release via pannexin-1 channels and possibly are not able to support autocrine, positive feedback loop through ATP interaction with P2X receptors, which stays beyond the scope of this study (Yip et al., 2008; Ledderose et al., 2014).

We have previously reported on defective PKC signaling in NSM2-deficient T-cells (Börtlein et al., 2018), which could provide an alternative explanation for the inability of these cells to upregulate mitochondria mass and OXPHOS upon antigenic stimulation. This is because NSM2 is a positive regulator of PKCζ and PKCθ which can phosphorylate CARMA1 upon co-stimulation. This initiates formation of multiprotein complex CARMA1-Bcl10-Malt1 (CBM) (Kingeter and Schaefer, 2008; Lopez et al., 2019). Failure to activate this complex could impair mTOR signaling, thereby preventing mitochondria biogenesis and proliferation in NSM2-deficient CD4^+^ T-cells.

NSM2 deficiency in Jurkat cells results in cellular cholesterol accumulation (Bortlein et al., 2019) which has been described to inhibit functions of some T-cell subtypes especially, for tumor-infiltrating lymphocytes (Ma et al., 2019). Increased cholesterol content can impair mitochondria respiration and thereby functionality (Balboa et al., 2017). Studies of energy metabolism in Niemann–Pick type C1-deficient cells demonstrated increased mitochondria cholesterol and deregulated mitochondria ATP transport to cytoplasm (Kennedy et al., 2014), indicating a possible link between NSM2 deficiency, accumulation of cellular cholesterol, and deregulated transport of mitochondria ATP.

Our data obtained on the importance of NSM2 in mitochondrial function have been generated using CD4 + T cells or Jurkat cells. It is, however, possible that NSM2 activity might be of different relevance in T-cell subsets depending on their vulnerability to deregulated OXPHOS or intrinsic levels of mitochondria activity. Impaired transition of T-cells to memory T cells after activation and defective clearance of pathogens in viral infections was found in a mouse model of human mitochondria diseases linked to disorders of oxidative phosphorylation (Tarasenko et al., 2017). Remarkably, mitochondria dysfunction differentially affected CD4^+^, CD8^+^, and T helper subsets depending on individual metabolic programs. However, it is beyond the scope of the present study, to evaluate the regulatory role of NSM2 in T cell subsets in a comprehensive manner.

As evident, T-cells with increased tonic signaling and metabolic activity in resting state can also become exhausted. Thus, increased basal Glut1 expression and glycolytic activity during quiescence can translate into T cell dysfunction, as particularly revealed for aged T cells or exhausted T cells in individuals having chronic viral infections (Schurich et al., 2016; Quinn et al., 2019). In turn, suppression of glycolytic activity in unstimulated CD8^+^ T cells can enhance memory and antitumor functions or even improve immune responses of aging T cells (Sukumar et al., 2013; Barzilai et al., 2016). We show here that sphingolipid metabolism at the plasma membrane communicates with cellular energy metabolism and that NSM2 deficiency drives quiescent CD4^+^ T cells into a state more characteristic for exhausted immune cells with enhanced tonic TCR signaling.

## Data Availability Statement

All datasets presented in this study are included in the article/Supplementary Material.

## Ethics Statement

The studies involving human participants were reviewed and approved by the Ethical Committee of the Medical Faculty of the University of Wuerzburg. Written informed consent for participation was not required for this study in accordance with the national legislation and the institutional requirements.

## Author Contributions

AS, SS-S, and EA: conceptualization, supervision. MD, AS, SR, and EA: methodology. MD: formal analysis. MD and EA: experimental investigation, writing. SS-S: funding acquisition. All authors contributed to the article and approved the submitted version.

## Conflict of Interest

The authors declare that the research was conducted in the absence of any commercial or financial relationships that could be construed as a potential conflict of interest.
