# Supplementary material for: Neutral Sphingomyelinase-2 (NSM 2) Controls T Cell Metabolic Homeostasis and Reprogramming During Activation
Source: Front Mol Biosci. 2020 Sep 4;7:217. doi: 10.3389/fmolb.2020.00217 (PMC7498697; doi:10.3389/fmolb.2020.00217)
Supplement: Supplementary file 1 [file Image_1.pdf]

Fig. S1

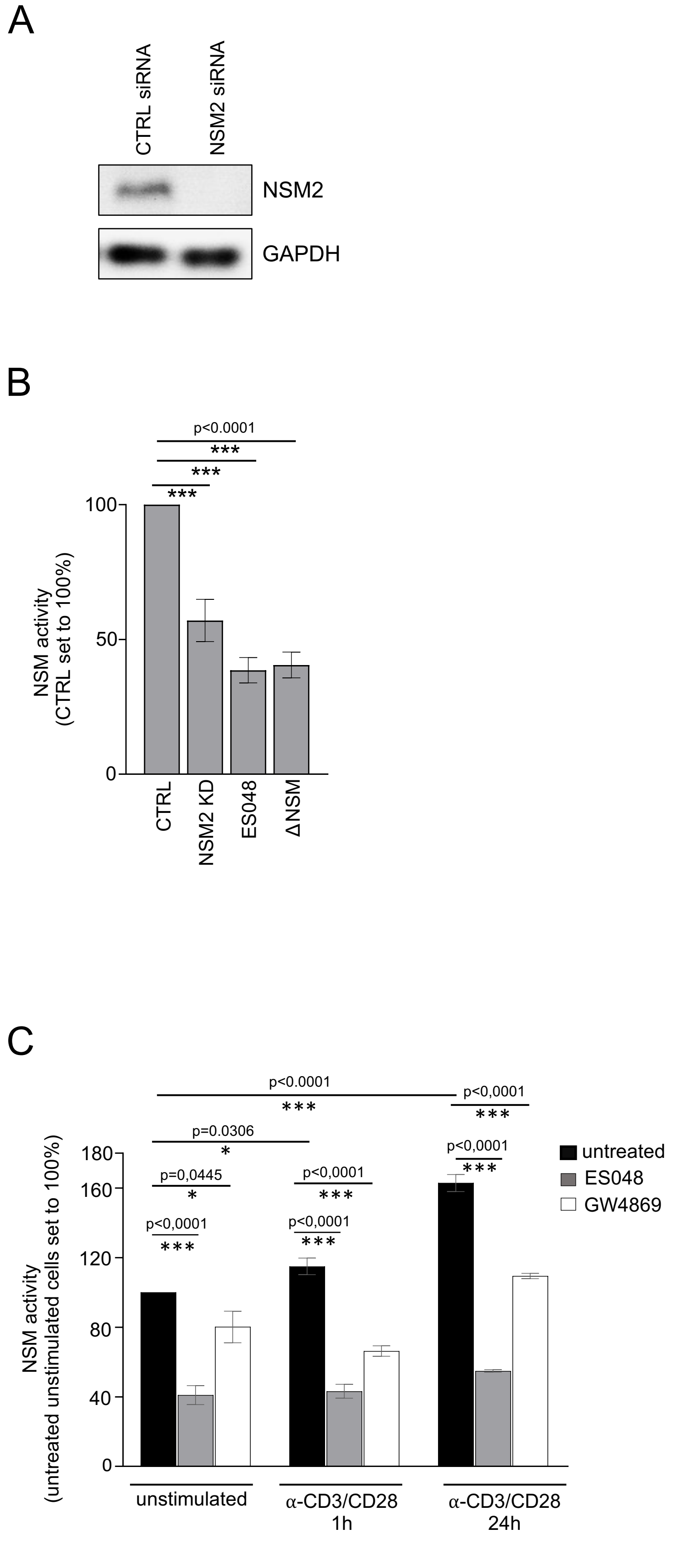
A
CTRL siRNA
NSM2 siRNA
NSM2
GAPDH
B
p<0.0001
***
***
***
NSM activity
(CTRL set to 100%)
100
50
0
CTRL
NSM2 KD
ES048
ΔNSM
C
p<0.0001
***
p<0,0001
***
p=0.0306
*
p<0,0001
***
p=0,0445
*
p<0,0001
***
p<0,0001
***
p<0,0001
***
untreated
ES048
GW4869
NSM activity
(untreated unstimulated cells set to 100%)
180
160
120
80
40
0
unstimulated
α-CD3/CD28
1h
α-CD3/CD28
24h

Fig. S2

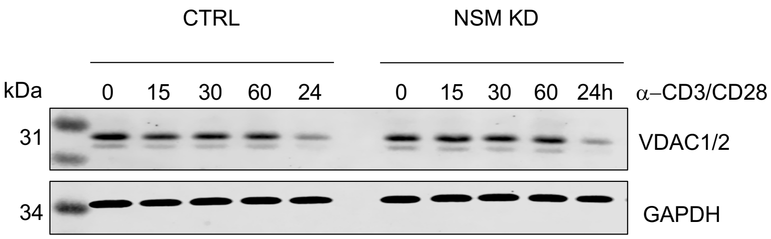
CTRL
NSM KD
kDa
0 15 30 60 24
0 15 30 60 24h
α–CD3/CD28
31
VDAC1/2
34
GAPDH

Fig. S3

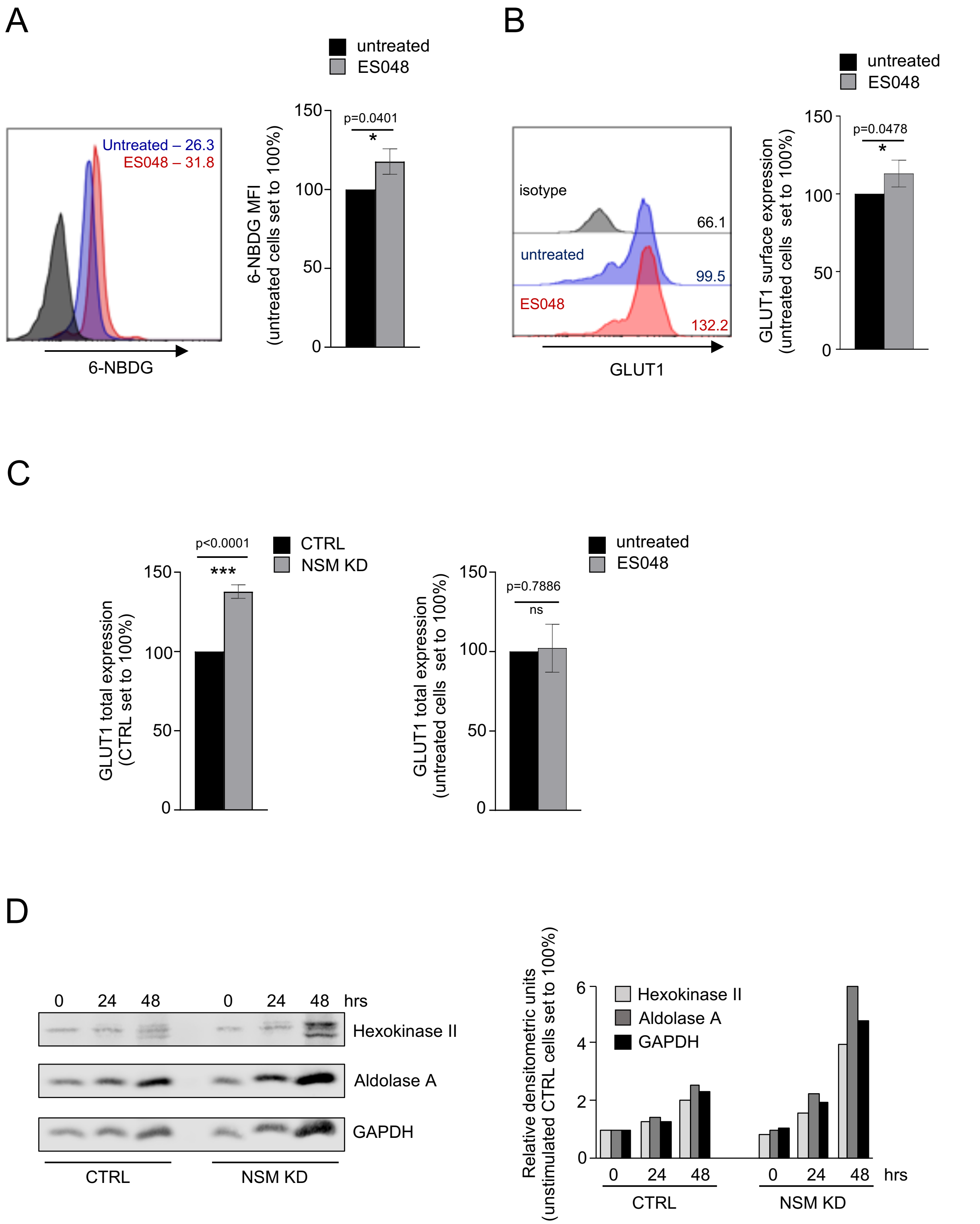
A
untreated
ES048
Untreated – 26.3
ES048 – 31.8
6-NBDG
150
100
50
0
p=0.0401
*
6-NBDG MFI
(untreated cells set to 100%)
B
untreated
ES048
isotype
untreated
ES048
66.1
99.5
132.2
GLUT1
p=0.0478
*
GLUT1 surface expression
(untreated cells set to 100%)
C
p<0.0001
***
CTRL
NSM KD
GLUT1 total expression
(CTRL set to 100%)
p=0.7886
ns
untreated
ES048
GLUT1 total expression
(untreated cells set to 100%)
D
0 24 48 0 24 48 hrs
Hexokinase II
Aldolase A
GAPDH
CTRL
NSM KD
Relative densitometric units
(unstimulated CTRL cells set to 100%)
Hexokinase II
Aldolase A
GAPDH
6
4
2
0
0 24 48 0 24 48 hrs
CTRL
NSM KD

Fig. S4

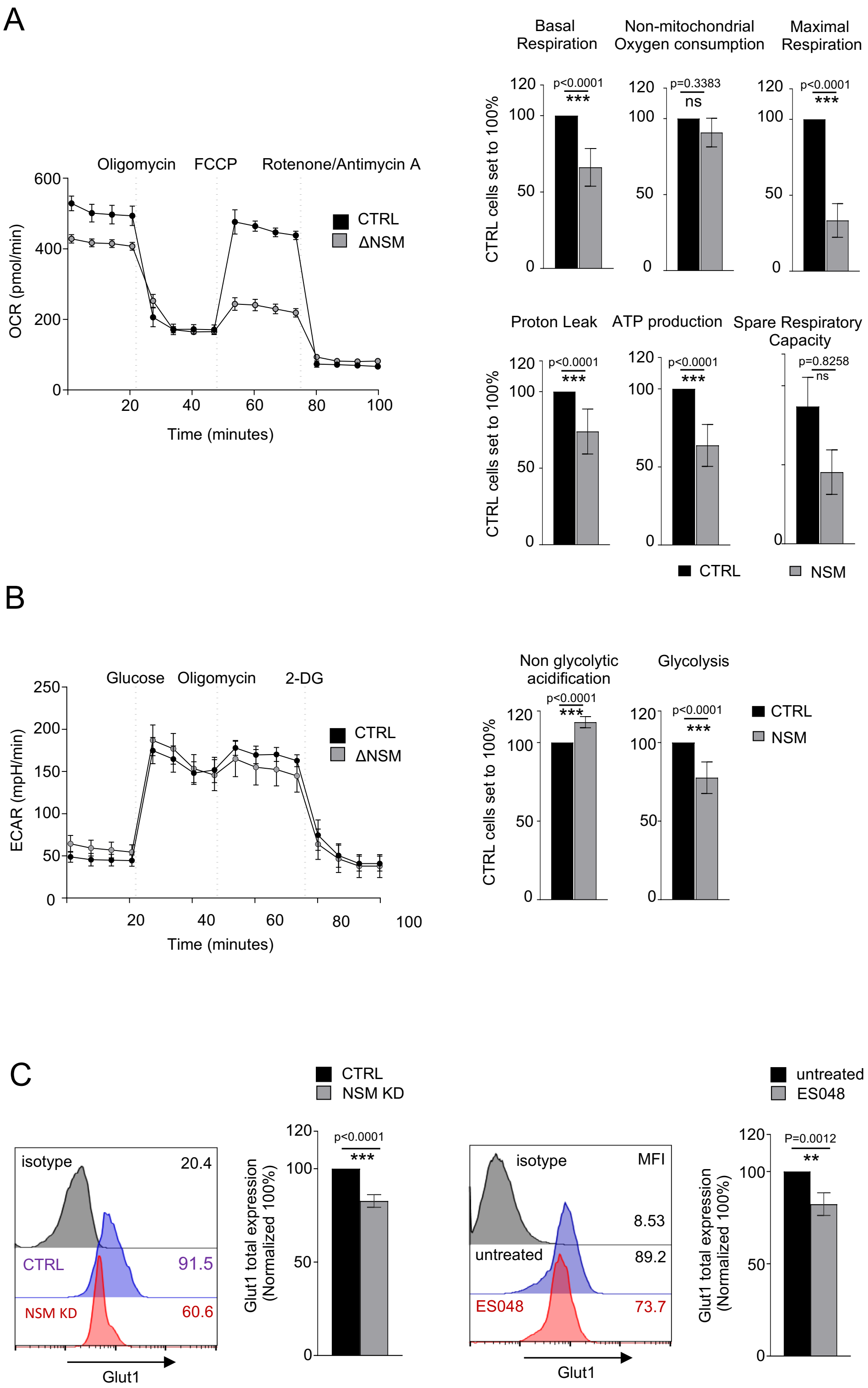
A
Oligomycin
FCCP
Rotenone/Antimycin A
OCR (pmol/min)
600
400
200
0
20
40
60
80
100
Time (minutes)
CTRL
ΔNSM
Basal Respiration
Non-mitochondrial Oxygen consumption
Maximal Respiration
Proton Leak
ATP production
Spare Respiratory Capacity
CTRL cells set to 100%
120
100
50
0
p<0.0001
***
p=0.3383
ns
p=0.8258
CTRL
NSM
B
Glucose
Oligomycin
2-DG
ECAR (mpH/min)
250
200
150
100
50
0
20
40
60
80
100
Time (minutes)
CTRL
ΔNSM
Non glycolytic acidification
Glycolysis
CTRL cells set to 100%
p<0.0001
***
CTRL
NSM
C
CTRL
NSM KD
isotype
20.4
CTRL
91.5
NSM KD
60.6
Glut1
Glut1 total expression (Normalized 100%)
120
100
50
0
p<0.0001
***
untreated
ES048
isotype
MFI
8.53
untreated
89.2
ES048
73.7
Glut1
Glut1 total expression (Normalized 100%)
P=0.0012
**

Fig. S5

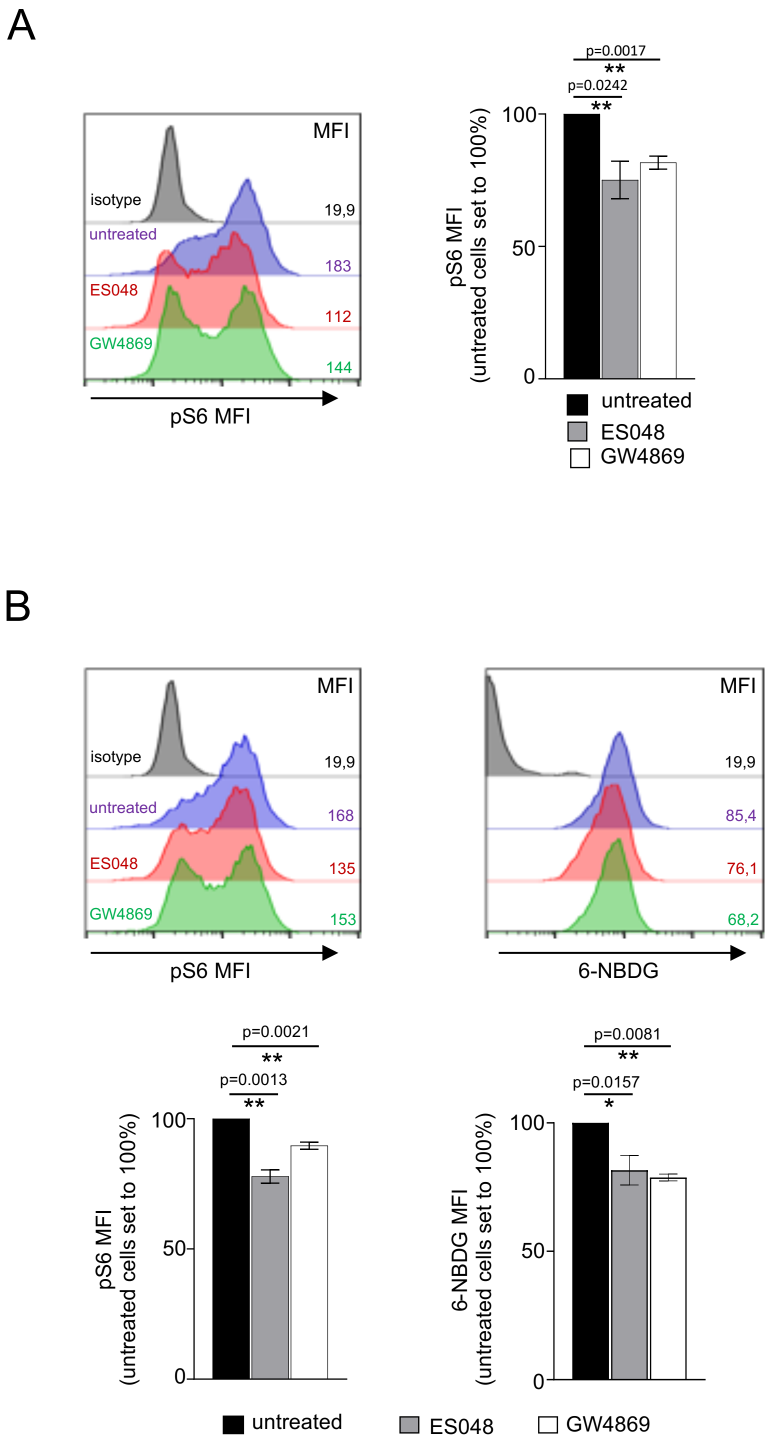
A
MFI
isotype
19,9
untreated
183
ES048
112
GW4869
144
pS6 MFI
p=0.0017
**
p=0.0242
**
100
50
0
pS6 MFI
(untreated cells set to 100%)
untreated
ES048
GW4869
B
MFI
isotype
19,9
untreated
168
ES048
135
GW4869
153
pS6 MFI
MFI
19,9
85,4
76,1
68,2
6-NBDG
p=0.0021
**
p=0.0013
**
100
50
0
pS6 MFI
(untreated cells set to 100%)
p=0.0081
**
p=0.0157
*
100
50
0
6-NBDG MFI
(untreated cells set to 100%)
untreated
ES048
GW4869

Fig. S6

A

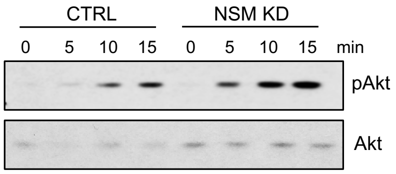
CTRL
NSM KD
0 5 10 15 0 5 10 15 min
pAkt
Akt

B

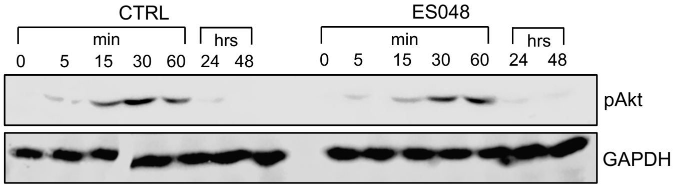
CTRL
ES048
min
hrs
0 5 15 30 60 24 48
0 5 15 30 60 24 48
pAkt
GAPDH

Fig. S7

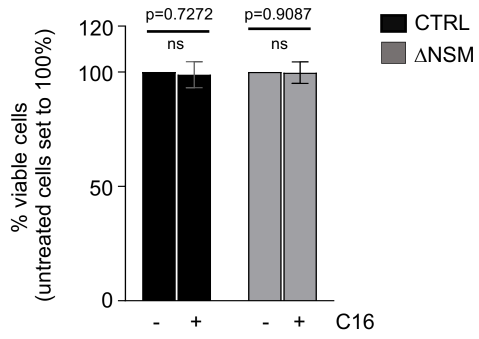
p=0.7272
p=0.9087
ns
ns
CTRL
ΔNSM
120
100
50
0
% viable cells
(untreated cells set to 100%)
-
+
-
+
C16

Fig. S8

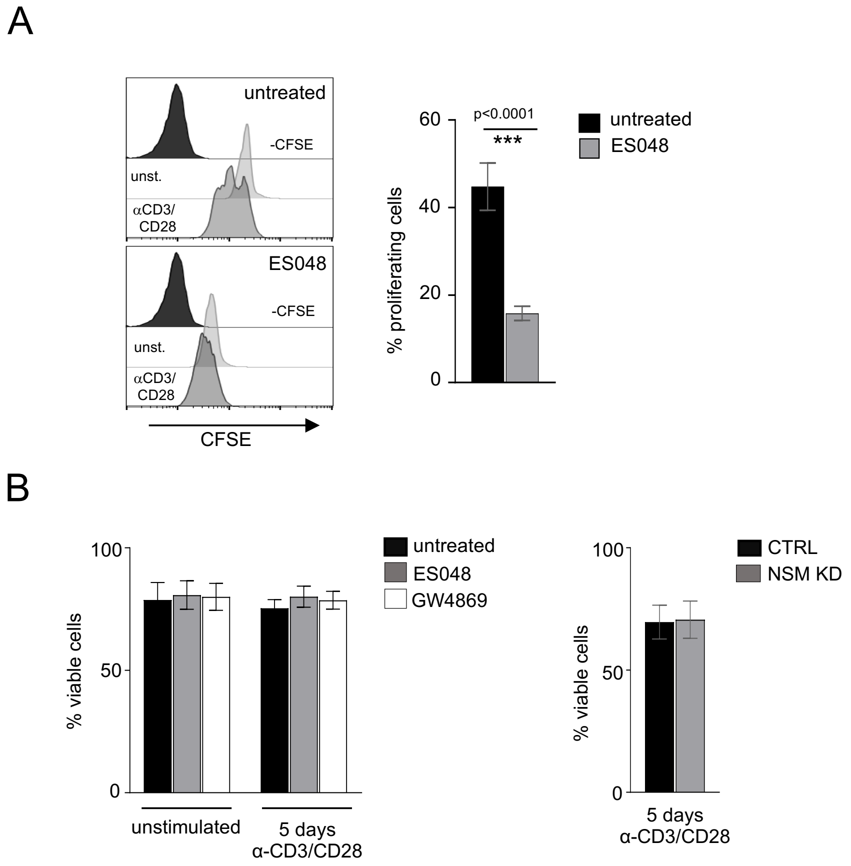
A
untreated
-CFSE
unst.
αCD3/
CD28
ES048
-CFSE
unst.
αCD3/
CD28
CFSE
p<0.0001
***
60
40
20
0
% proliferating cells
untreated
ES048
B
100
50
0
% viable cells
untreated
ES048
GW4869
unstimulated
5 days
α-CD3/CD28
100
50
0
% viable cells
CTRL
NSM KD
5 days
α-CD3/CD28

Supplementary figures

Figure S1: Downregulation of neutral sphingomyelinase 2 expression and activity in primary human $CD4^+$ T-cells

A. NSM2 mRNA expression was analyzed by RT-PCR in T cells 5 days post-nucleofection with control (CTRL) or NSM2 specific siRNA.

B. NSM activity was measured in ES048 treated cells, NSM2 siRNA transfected (NSM KD) and in NSM2 knock-down Jurkat cells (ΔNSM). Reduction rates in each group of cells were normalized against according control cells and untreated, siRNA transfected or CTRL sgRNA expressing cells (CTRL) are set to 100%.

C. $CD4^+$ T cells were pretreated or not for 2 hours with 1, 5 μM ES048 or 1 μM GW4869 and NSM activity was measured in lysates from unstimulated or cells after stimulation with α-CD3/CD28 for 1 or 24 hours.

Mean values with standard deviations are shown for five (B) or three (C) independent experiments. p-value is shown on the top of significant differences (marked with asterisks).

Figure S2: VDAC1 expression is regulated NSM2 independently

Western blot analysis of VDAC1/2 expression in $CD4^+$ CTRL and NSM KD T-cells. Cells were co-stimulated with α-CD3/CD28 for different time points.

Figure S3: Glucose uptake and Glut1 expression is enhanced in NSM2 deficient resting T-cells

A and B. Glucose analogue 6-NBDG uptake (A) and Glut1 surface expression (B) were measured in untreated and ES048 pretreated cells. Measurements from three independent experiments were normalized against co-stimulated untreated or CTRL cells which are set to 100%.

C. Glut1 total expression levels were analyzed by flow cytometry in unstimulated CTRL and NSM2 KD (left graph) or untreated and ES048 treated (right graph) $CD4^+$ T-cells. Measurements were normalized against CTRL or untreated cells set to 100%.

Mean values with standard deviations are shown. p-values are shown on the top of significant (marked with asterisks) or not significant differences (ns).

D. Western blot analysis of glycolytic protein expression in CTRL and NSM KD $CD4^+$ T cells left unstimulated or stimulated with α-CD3/CD28 for 24 and 48 hours. Densitometric analysis of Western blot is shown in the right graph.

Figure S4: NSM2 activity regulates oxidative phosphorylation and Glut1 expression in activated T-cells

A and B. Mitochondrial stress test (A) and glycolytic stress test (B) were performed in CTRL and ΔNSM Jurkat cells. Oxygen consumption Rates (OCR) and Extracellular Acidification Rates (ECAR) was measured using a XF96 Seahorse metabolic flux analyzer. Representative graphs are shown in the left graphs. Analysis of basal respiration, non-mitochondrial oxygen consumption, maximal respiration, proton leak, ATP production, spare respiratory capacity, non-glycolytic acidification and glycolysis from three independent experiments is shown (right graphs).

C. CTRL and NSM2 KD cells (left graphs) or $CD4^+$ T-cells left untreated and ES048 pretreated (right graphs) were stimulated with α-CD3/CD28 for 24 hours and Glut1 total expression levels were analyzed by flow cytometry. Measurements were normalized against CTRL or untreated cells set to 100%.

Mean values with standard deviations are shown. p-values are shown on the top of significant (marked with asterisks).

Figure S5: NSM2 activity in TCR stimulated CD4$^+$ T cells promotes glucose uptake and mTOR pathway activation

A and B. CD4$^+$ T cells were left untreated or NSM inhibitors: ES048 and GW4869, were added 2 hours before (A) or 2 hours after (B) α-CD3/CD28 stimulation. Phosphorylation of S6 and glucose analogue 6-NBDG uptake was analyzed by flow cytometry. Representative flow cytometry histograms. Measurements of three independent experiments were normalized against untreated cells set to 100%. Mean values with standard deviations are shown. p-values are shown on the top of significant differences (marked with asterisks).

Figure S6: Akt kinase phosphorylation in TCR/CD28 co-stimulated T-cells is NSM2 independent

CTRL or NSM KD cells (A) or CD4$^+$ T-cells left untreated and ES048 pretreated for 2 hours (B) were α-CD3/CD28 co-stimulated for different time points and phosphorylated Akt was analyzed in Western blot.

Figure S7: Exogeneous supplemented C16 ceramides are not affecting viability of Jurkat cells

CTRL and ΔNSM Jurkat cells were left untreated or incubated overnight in cell culture medium supplemented with 25 μM C16 ceramides. Cell viability was estimated by flow cytometry excluding propidium iodide (PI) and Annexin V positive cells. Measurements of three independent experiments were normalized against untreated cells set to 100%. Mean values with standard deviations are shown. p-values are shown at the top of non-significant differences (marked with ns).

Figure S8: NSM2 is not required for T cell survival.

A. CD4$^+$ T cells were left untreated or NSM inhibitor ES048 was added 2 hours before CFSE labelling and α-CD3/CD28 stimulation. Flow cytometry analysis of CFSE fluorescence was performed 5 days after stimulation.

B. CD4$^+$ T-cells were left untreated and ES048 or GW4869 pretreated (left graph) or CTRL and NSM2 KD cells (right graph) were left unstimulated or stimulated with α-CD3/CD28 for 5 days. Cell viability was estimated by flow cytometry excluding propidium iodide (PI) positive cells.

Mean values with standard deviations are shown. p-values are shown at the top of significant differences (marked with asterisks).
